# Advancing the Measurement of Executive Functioning in Pediatric Chronic Pain

**DOI:** 10.3390/children8080630

**Published:** 2021-07-24

**Authors:** Emily A. Beckmann, Kristen E. Jastrowski Mano

**Affiliations:** Department of Psychology, University of Cincinnati, Cincinnati, OH 45221, USA; manokn@ucmail.uc.edu

**Keywords:** executive functioning, cognition, measurement, pediatric, chronic pain

## Abstract

Youth with chronic pain often report executive functioning difficulties, many of which have been linked to poor treatment adherence and health-related quality of life in adults with chronic pain, as well as in other pediatric chronic health populations. Despite the extensive implications for functional impairment, executive functioning remains understudied in pediatric chronic pain. Measurement approaches have lacked clear theoretical guidance, resulting in only some domains of executive functioning being investigated. To date, the methods used to measure executive functioning have been inconsistent, ranging from self-report measures of everyday executive functioning in home and school contexts to standardized neuropsychological tests. We argue for enhanced measure validation efforts and increased clarity in the approaches chosen to measure executive functioning in pediatric chronic pain to better guide research efforts in this area, thus yielding clearer clinical implications.

## 1. Introduction

Pediatric chronic pain is a prevalent condition affecting nearly 23 percent of young people [1]. It is well documented that pain in youth is often accompanied by a variety of physical [2,3,4], emotional [5,6,7], and social impairments [8,9]. Less clear is the nature of cognitive impairments in this population, despite numerous studies identifying executive functioning (EF) deficits in adults with chronic pain [10,11,12,13] The literature in pediatric chronic pain is premature; however, youth with chronic pain report difficulty with inhibition, cognitive flexibility, and working memory [14,15,16,17,18,19], abilities collectively labeled as executive functioning.

### 1.1. Pediatric Chronic Pain

Pediatric chronic pain is complex, involving dynamic interactions among myriad biological, psychological, and socio-cultural influences [1,2,3,4,5,6,7]. Chronic pain symptoms can be persistent, where pain is present without relief, or recurrent, in which the pain subsides and returns episodically. For some children and adolescents, chronic pain intensity is mild to moderate, for others it is severe [20]. Chronic pain can be experienced in the context of another medical condition, such as sickle cell disease [21] or inflammatory bowel disease [22]. However, pediatric chronic pain also manifests on its own. Common primary pain disorders in young people include chronic back pain, headache, and Complex Regional Pain Syndrome (CRPS) [7,23].

### 1.2. Executive Functioning

**Figure 1:**
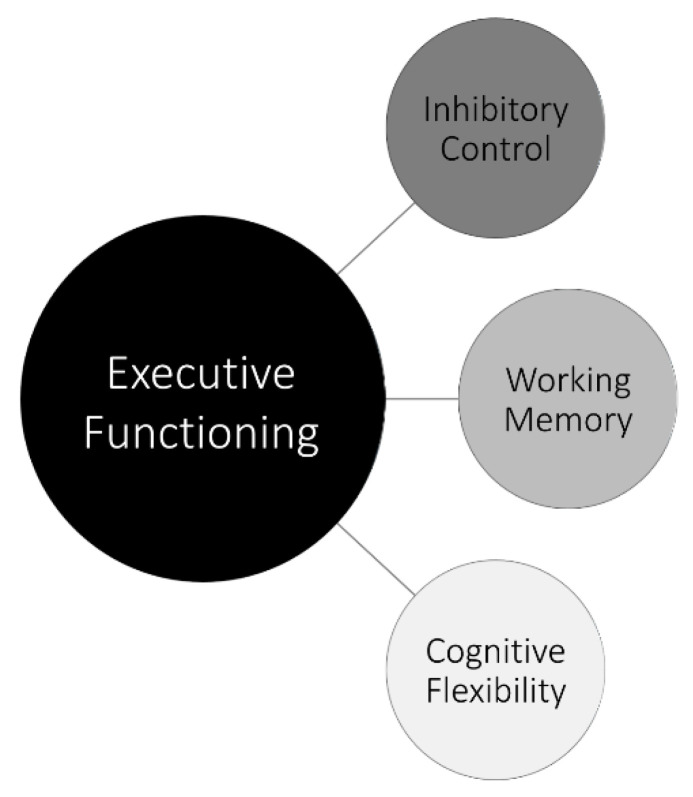
An illustration of the three core executive functions [26]. This theory of executive functioning proposes that there are three related, but distinct, aspects of executive functioning: inhibitory control, working memory (i.e., updating), and cognitive flexibility (i.e., shifting).

Executive functioning (EF) is defined as a collection of higher-order cognitive processes underlying goal-directed behavior and is often referred to as the “conductor” that controls, organizes, and directs cognitive activity, emotional responses, and behavior [2,3,4,5,6,7,8,9,10,11,12,13,14,15,16,17,18,19,20,21,22,23,24,25,26]. EF deficits may manifest in greater difficulty paying attention, regulating emotions, and completing tasks independently. Though there is variation in the operationalization and choice of tasks when measuring EF, current theoretical and empirical research agrees that inhibitory control, working memory, and cognitive flexibility are the core components of EF [17,23,27] (Figure 1) and are useful when studying EF in children [28,29].

Inhibitory control (IC) is characterized by one’s ability to exhibit mental control over their thoughts, feelings, and behaviors in order to override external distractors and rather engage with or in the more appropriate or necessary task [25]. IC is a critical skill required for on-task behavior in the face of distractions (e.g., studying for a test while ignoring notifications appearing on their phone or computer). WM necessitates the maintenance and manipulation of information in the mind [30]. WM is a process that is required to understand written and spoken language, perform mental mathematics, as well as reason or problem solve. For young people, working memory difficulties might make it challenging to remember a list of instructions for an assignment given by a parent, teacher, or coach. Lastly, cognitive flexibility involves the ability to change perspectives both spatially as well as in social contexts [19]. For children and adolescents, a deficit in cognitive flexibility might present as difficulty in altering expectations and/or coping with changes in routines or schedules.

Unsurprisingly, difficulties related to the processes of inhibitory control, working memory, and cognitive flexibility often result in marked functional impairment [25]. EF difficulties have been linked to challenges in daily functioning, such as difficulty staying on task, selectively attending to relevant information, planning, organizing, or completing tasks, and difficulty regulating emotions as well as following directions [31].

### 1.3. EF and Functional Impairment in Pediatric Chronic Pain

Understanding the exact nature of EF deficits in pediatric chronic pain has direct implications for functional impairment [16]. For youth with chronic pain, impairment is often noticed in the context of school. EF has been shown to be a critical mechanism in explaining difficulties in the context of school performance within the pediatric chronic pain population [14]. EF impairments have serious implications for tasks that are reliant on cognitive flexibility. For example, a child with EF deficits may have difficulty focusing during class, taking notes, completing homework, and/or preparing for a quiz or test [31]. Not only does school impairment involve difficulties in academic functioning, but it involves social functioning as well. EF deficits have been linked to strained peer relationships as well as difficulties making and keeping friends [19,32], which are critical components of the school experience for children and adolescents.

EF is an essential part of treatment adherence, as youth with chronic medical conditions often need to make difficult and important decisions regarding their treatment and practice flexibility and adaptability when their treatment changes [33,34,35]. Research has identified a variety of pediatric chronic health conditions (e.g., type-1 diabetes, cystic fibrosis, asthma, and epilepsy) in which EF deficits had a negative impact on treatment adherence. Treatment non-adherence can be particularly problematic because it has been associated with both short term and long-term adverse health outcomes [36,37]. Although it has been posited that EF might be a factor impacting treatment adherence in pediatric chronic pain, this has yet to be examined.

Youth with chronic pain often experience emotional impairment. Specifically, pediatric chronic pain has been linked to a variety of mental health conditions, such as anxiety and depressive disorders [5,38]. Research suggests that high rates of anxiety and depression in pediatric chronic pain could be explained by biological (e.g., genes, hormones, heightened alarm response), cognitive (e.g., attentional biases), and behavioral factors (e.g., sleep difficulties) [6,39,40,41]. Moreover, EF impairments have been linked to psychopathology. The literature on anxiety has identified impairments in the EF domains of cognitive flexibility [42,43,44], inhibition [27,45,46], and working memory [47]. In terms of depression and EF, meta-analytic evidence suggests that individuals with major depression also experience difficulties in cognitive flexibility, inhibition, and working memory [48].

## 2. Measurement of EF in Pediatric Chronic Pain

Given the complex, multifaceted nature of EF it is perhaps not surprising that those studying cognitive difficulties in the context of pediatric chronic pain have utilized a variety of measurement approaches (Table 1). Some have cast a wide net, broadly gauging EF across various domains [15,16,49,50], whereas others have focused more narrowly on individual components of EF, such as working memory [17,23,51]. In some cases, researchers have utilized measures of EF without explicitly referring to EF [17,51]. It is important to note that existing EF research in pediatric pain has varied greatly in terms of the research question, EF domains, and sample characteristics (e.g., pain condition). Findings have been inconsistent even among studies employing similar samples, highlighting the potential role that measurement methods may play in examinations of EF in pediatric chronic pain.

**Figure 2:**
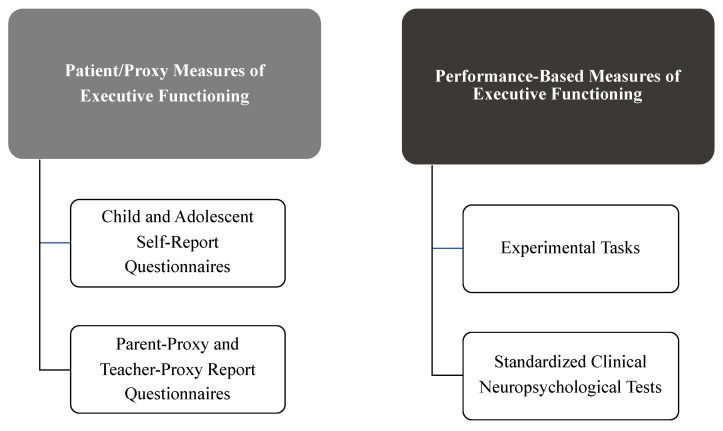
Methods of measuring executive functioning in youth with chronic pain.

Measurement methods generally fall into the following categories: youth self-report, parent-proxy report, experimental performance-based tasks, and standardized neuropsychological tests (often including intelligence quotient (IQ) and academic achievement tests as part of the assessment battery) (Figure 2).

### 2.1. Youth Self-Report and Parent-Proxy Report

To date, only one youth self-report and parent-proxy EF questionnaire has been utilized in research measuring EF in youth with chronic pain. Specifically, the Behavior Rating Inventory of Executive Function [24] (BRIEF-2) has been utilized to assess EF deficits in older children and adolescents with chronic pain (ages 11–18). The BRIEF-2 measures how children and adolescents (or their parents) perceive their own “everyday” EF in home and school environments. The BRIEF-2 youth self-report includes seven subscales: Inhibit, Self-Monitor, Shift, Emotional Control, Task Completion, Working Memory, and Plan/Organize. Inhibit measures assess inhibitory control and impulsivity. Self-Monitor appraises the young person’s awareness of the impact of their behavior on other individuals as well as the outcomes of those behaviors. It captures the level to which the individual is able to detect or notice how their behavior is received by others as well as how their behavior compares with expectations of others as to how they should behave in the particular context that they are in. Shift assesses the ability to “shift” or go from one activity to another as appropriate. Emotional Control assesses the degree to which EF impacts the young person’s emotional expression and measures their ability to control or alter their emotional responses. Task Completion assesses the young person’s ability to complete tasks in a timely manner as well as if EF impacts this process. Working Memory assesses the young person’s perceived ability to maintain information in their minds in the short term so that they are able to complete particular tasks. Lastly, Plan/Organize assesses the young person’s perceived ability to anticipate future events and work proactively to achieve future goals (Plan) and to appropriately order information or materials in the context of learning or communicating (Organize). The parent-proxy report of the BRIEF-2 includes the same subscales as the youth self-report (i.e., Inhibit, Self-Monitor, Shift, Emotional Control, Working Memory, and Plan/Organize), but in place of Task Completion, it includes a scale assessing the ability to start a task and work on it independently while utilizing problem-solving skills (Initiate). Studies using the BRIEF-2 [16,19,23] have generally found that youth with pediatric pain (and/or their parents) report worse EF relative to healthy controls across a number of domains, including working memory and cognitive flexibility (see Table 1 for details).

### 2.2. Performance-Based Experimental EF Tasks

A variety of performance-based experimental tasks have been used in studies of EF in youth with chronic pain, including a computerized anti-saccade task, Children’s Paced Auditory Serial Addition (CHIPASAT), the Rey Complex Figure Test (RCFT), and the Delis-Kaplan Executive Function System (D-KEFS).

Anti-saccade tasks [18] assess inhibition by requiring respondents to inhibit innate or automatic responses to task stimuli. The color-word test [18] assesses both cognitive flexibility (i.e., the ability to shift cognitive set) and the inhibition of a habituated response in favor of a novel or unusual one. The specific color-word test used by Verhoeven and colleagues [18] involved three cards with varying instructions. The first card (words) displayed 100 color names (blue, green, red, and yellow) written in black ink. Participants were instructed to read the words as quickly as they could. The second card displayed 100 color blocks (color) (blue, green, red, and yellow). In this condition participants were instructed to identify each color block as quickly as possible. In the last condition, the card displayed color words (blue, green, red, and yellow) printed in differing colors (interference). Participants were instructed to identify the ink color and suppress their automatic response to read the word. More recently, Turner and colleagues [50] used several performance-based experimental EF tasks (See Table 1) and found that youth with chronic pain had significantly lower scores on several tests of working memory, divided attention, inhibition, and flexibility.

### 2.3. Neuropsychological Tests

Neuropsychological tests are tasks designed to assess a particular psychological function that has been linked to a specific neurological pathway or brain structure. One battery of neuropsychological tests used to assess EF in pediatric chronic pain was the TEA-Ch battery [15], which consists of five separate tasks that assess the ability to selectively attend, sustain attention, divide attention, as well as switch attention. Among youth with functional abdominal pain, selective attention abilities were inversely associated with pain coping [15].

The Wide Range Assessment of Memory and Learning (WRAML-2) has also been used in one study of EF in pediatric chronic pain [19]. The WRAML-2 is a neuropsychological test of memory functioning. It assesses both immediate memory as well as delayed memory abilities along with learning. The WRAML-2 has two verbal, two visual, and two attention/concentration subscales, which yield three indices: Verbal Memory, Visual Memory, and Attention/Concentration. Scores on the Verbal Memory Index are related to one’s ability to learn and recall both relevant and less relevant verbal information. Scores on the Visual Memory Index reflect learning and recall ability of relevant information in pictorial and design contexts. Lastly, scores on the Attention/Concentration Index are demonstrative of the young person’s ability to learn and recall less meaningful information presented in a sequential manner. Weiss and colleagues [19] tested verbal working memory and symbolic working memory, but only reported composite working memory scores. Five percent of the sample scored 1.5 SDs below the normative mean.

### 2.4. IQ and Achievement Tests

IQ and Achievement tests have also been used to measure specific domains of EF in pediatric chronic pain. Cruz and colleagues [24] used the Wechsler Intelligence Scales for Children (WISC), an IQ test for children and adolescents, in order to measure cognitive functioning in youth with chronic pain. They focused on WM subtests as well as processing speed. Approximately one-third (36%) of the sample exhibited risk/impaired attention/working memory composite scores. Greenly and colleagues [48] used the Kaufman Brief Intelligence Test (K-BIT) and focused on non-verbal subtests with the aim of better understanding cognitive flexibility. They also utilized several measures of achievement (Wide Range Achievement Test (WRAT); Grey Oral Reading Test (GORT); Test of Written Language (TOWL) in order to determine if the demonstrated cognitive difficulties were associated with poorer academic achievement. Taken together, though youth with pediatric chronic pain generally outperform healthy controls on IQ and achievement measures, they exhibit a notable weakness in the specific EF domain of WM [23,31].

## 3. Key Challenges for Future Research

Despite an increased interest in EF in pediatric populations, numerous challenges remain in understanding EF in pediatric chronic pain. To date, although findings have been fairly consistent in showing a link between EF and impairment in chronic pain [16], research in this area has proceeded in the relative absence of a guiding theoretical framework. A piecemeal approach is unnecessary given the rich infrastructure of EF in related fields. For example, in clinical neuropsychology, much support has been garnered for a three-dimensional model of EF (see Figure 1) that includes three unique, but related components, namely inhibitory control, working memory, and cognitive flexibility [26,52]. Whether this model, relative to others, is relevant to pediatric pain remains an empirical question, but is a critical next step in advancing work in this burgeoning area of inquiry.

Another limiting factor inhibiting research in this area is a lack of validation efforts establishing the appropriateness of existing EF measures for use in pediatric chronic pain and without establishing the psychometric properties of available EF measures for use with pediatric chronic pain patients. Although extant questionnaires, such as the BRIEF-2, and neuropsychological tests of EF domains have been validated in child psychiatric populations (e.g., Attention Deficit Hyperactivity Disorder (ADHD), Autism Spectrum Disorder (ASD)), it is unknown whether these measures adequately capture the nature of EF—and are helpful in predicting functional impairment—in youth with chronic pain. Therefore, research is needed to solidify both the content of EF measures as well as the construct validity (i.e., the convergent and discriminant validity) of experimental measures among youth with chronic pain. Without establishing the psychometric properties of existing—or newly developed—EF measures in pediatric pain populations, conclusions drawn from EF studies remain equivocal.

Another potential challenge in understanding EF in pediatric chronic pain is the inherent variability across chronic pain conditions. Thus, individual study findings may not widely generalize. Previous studies examining EF in pediatric chronic pain have included small samples (ranging from N = 13 to 57) of youth between the ages of 8 and 19 with a variety of primary pain conditions (additional sample characteristics can be viewed in Table 1). For example, studies have assessed EF in youth with Complex Regional Pain Syndrome (CRPS), musculoskeletal pain, functional abdominal pain, and headaches. Other studies have focused on medical conditions in which pain is a common symptom (e.g., sickle cell disease). Moreover, most have recruited primarily from outpatient pediatric pain clinics, with few including participants from inpatient pain programs or community samples. To the degree that this creates range restriction in our measures of functional disability (e.g., pain-related physical impairment), important associations between EF and functional outcomes may go undetected. Taken together, small sample sizes, heterogenous sample characteristics, and a restricted focus on outpatients demands additional research to determine whether a particular measurement method is equally appropriate across diverse pediatric chronic pain populations.

Research on EF measurement—regardless of clinical population—has consistently found low magnitude correlations between different methods of measuring EF [50]. For example, self-reports of EF (e.g., BRIEF-2) are often weakly related to experimental performance-based EF measures (e.g., Go/No-Go [53], Wisconsin Card Sort Task [50]). This is partially due to the fact that questionnaire measures of EF tend to focus on “everyday” manifestations of EF impairment, as opposed to EF in a context-free, optimally controlled environment. This is because most performance-based and neuropsychological measures are designed to evaluate one’s EF performance in a relatively controlled (e.g., quiet, relatively distraction free) environment to determine one’s optimal performance. This distinction is important, as no one method wholly captures EF; rather, each method provides complementary information, or different pieces of the puzzle. By extension, it is plausible that different assessment methods differentially predict important clinical correlates (e.g., treatment adherence) and functional impairment. This remains an empirical question, of course, but such research is important to establish the criterion validity of EF measures used in pediatric chronic pain. Similarly, different measurement methods may provide unique information to clinicians. For example, how young people perceive their own EF difficulties at school and home may provide clinicians with specific, concrete treatment targets, whereas standardized neuropsychological EF tests would provide a clearer understanding of the child’s relative strengths and weaknesses across EF domains relative to age- and gender-matched norms. Thus, a comprehensive, multi-method EF measurement approach is needed.

Multi-informant measurement approaches are also important, particularly if questionnaire methods are used exclusively. As discussed earlier, to date, only the BRIEF-2 has been used in pediatric chronic pain EF research. Despite consistent evidence of low inter-rater reliability of youth and parent-report measures in pediatric and child clinical psychology research (e.g., [38,54,55]), and the availability of BRIEF-2 youth, parent, and teacher-report forms, most studies have only taken into account the ratings from one source.

Including multiple informants in the assessment of EF in youth with chronic pain would bolster the evidence for impairment in particular domains of EF if similar difficulties were reported by multiple sources. Unlike parents and teachers, youth with chronic pain experience the direct impact of executive dysfunction on their daily life. In pediatric chronic pain, it has been established that the youth self-report is often a useful method of assessment [53,54,55]. For example, studies have determined that children and adolescents can accurately report on their pain intensity, quality of life, and comorbid psychopathology [56,57]. Despite such evidence, a valid and reliable measurement of EF by youth self-report has yet to be established in the literature.

From a developmental perspective, children and adolescents have varying introspective abilities. EF difficulties may be particularly challenging for some young people to accurately report [58]. To address this potential limitation, parent and teacher reports of EF are also important to consider as each can uniquely report on the challenges young people experience in their daily interactions in different environments (e.g., home, school). Parents are able to provide a unique perspective on the manifestation of EF difficulties that their child might not fully recognize. Indeed, research in other clinical populations (e.g., ADHD, traumatic brain injury) has demonstrated that youth self-report and parent behavior rating scales of EF are only weakly correlated [58], suggesting that integrated multi-informant ratings are necessary to precisely capture EF impairment. Because teachers interact with children of varying abilities, teacher ratings of youth EF provide additional insights into the impact of executive dysfunction on a child’s school-related functioning relative to age-matched peers [59,60,61]. Additionally, relative to home, school settings allow for EF behaviors to be assessed in a more structured, consistent environment that places more cognitive demands on the child. Despite the availability of teacher rating scales of EF for youth, studies have not yet utilized teacher behavior ratings in the measurement of EF in pediatric chronic pain. Including teacher reports in future studies evaluating EF in pediatric pain would bolster the ecological validity of EF measures. Further, given that EF deficits in pediatric chronic pain likely vary from child to child and across different settings and situational demands (e.g., expected behavior at school relative to home), teacher reports may provide contextual specificity when identifying areas of concern.

Though multifaceted definitions of EF have generally been referenced, the measurement methods used in pediatric pain research have rarely examined multiple domains of EF in a single study. For example, Verhoeven and colleagues [18] measured Inhibitory Control and WM, however these are only two of several EF domains. Turner and colleagues [50] included multiple measurement methods, and their results demonstrated concordance between performance-based tasks and behavior ratings. However, the study sample was small (*n* = 26 youth with chronic pain), thus generalizability is limited. Without careful examinations of multiple EF domains, it will be challenging to accurately determine the domains of EF that are most impacted in pediatric chronic pain. Therefore, future studies should also incorporate multiple measurement methods with larger samples—both community- and clinic-based—in a systematic and theory-informed manner.

## 4. Clinical Utility of EF Measures

Once researchers have (1) established the reliability and validity of EF measures for use in pediatric chronic pain, (2) determined which domains of EF are most impaired in pediatric chronic pain patients, and (3) identified the specific measures that best distinguish between youth with chronic pain and healthy controls, then we must (4) establish which EF measures best predict key clinical and functional outcomes in youth with chronic pain. To date, research that has included both a pediatric chronic pain sample and healthy controls has focused—rightly so—on establishing group differences. This is a helpful starting off point, and much more in this area is necessary. However, even if relatively consistent group differences emerge (i.e., youth with chronic pain exhibit elevated EF dysfunction in certain EF domains), elevated scores alone do not guide clinical practice and do not necessarily shed light on the degree to which EF deficits are associated with clinical outcomes. Rather, we need to establish which domains of EF are specifically linked to particular areas of functional impairment (e.g., school functioning) and clinical outcomes (e.g., treatment adherence, response to treatment). It remains unclear whether there are unique EF risk profiles associated with certain areas of functional impairment.

## 5. Conclusions

Youth with chronic pain often report cognitive difficulties, specifically when it comes to inhibition, cognitive flexibility, and working memory [14,15,16,17,58]. Current measurement approaches are hindering our understanding of executive functioning in pediatric chronic pain. In the limited literature on EF in pediatric chronic pain, the domains measured and the methods used are inconsistent. Such measurement discrepancies demonstrate the need for consistency and specificity in the definition of EF within this population. Research supports taking a multidimensional approach to the study of EF [47] as opposed to a fragmented, compartmentalized approach. Future research efforts also need to determine the degree to which existing clinical neuropsychological models of EF are appropriate for application to pediatric chronic pain.

## Figures and Tables

**Table 1:** Measurement of EF in Pediatric Chronic Pain.

Study	EF Domains	Sample	Measurement Method (s)	Summary of Results
Cruz, O’Reilly, Slomine, and Salorio (2011)	Attention, WM	17 youths (9–18 years old) with CRPS-1. Inpatient rehabilitation program.	Youth self-report (BRIEF-2); IQ (WISC)	36% showed at risk/impaired attention and/or working memory scores (BRIEF). Few participants demonstrated impairment on the WISC.
Greenly, Bennett, Cox, and Poole (2008) ^1^	WM	57 youths (8–18 years old) with chronic pain. Heterogenous outpatient pain clinic sample.	IQ (K-BIT); Academic Achievement (WIAT, WRAT, GORT, TOWL)	Chronic pain patients scored higher in general intelligence and academic achievement. CP patients demonstrated high scores on all IQ subtests compared to controls with the exception of WM.
Hocking, Barnes, Shaw, Lochman, Madan-Swain, and Saeed (2011)	Selective Attention, Inhibition, Self-Monitoring, Shifting, Emotional Control, Task Completion, WM, Planning and Organization	44 youths with FAP and parents (6–18 years old). Tertiary care pediatric medical center.	Neuropsychological test (TEA-Ch); Parent-proxy report (BRIEF-P)	Significant relationship between selective attention and coping with pain. A total of 27% of the sample was in the clinical range for the BRIEF global executive composite score. Mean performance on the TEA-Ch attention tests was in the borderline-to-low average range.
Jastrowski Mano, Beckmann, Fussner, and Kashikar-Zuck (2020)	Inhibition, Self-Monitoring, Shifting, Emotional Control, Task Completion, WM, Planning and Organization	60 adolescents (30 with musculoskeletal pain, 30 healthy controls) (12–17 years old). Inpatient rehabilitation and outpatient pain clinic.	Youth self-report (BRIEF-2)	Chronic pain group scored above the clinical risk cut off for WM (52%), inhibition (45%), and cognitive flexibility (38%). EF was significantly related to functional disability.
Ludwig, Sil, Khowaja, Cohen, and Dampier (2018)	Inhibition, Shifting, Emotional Control, WM, Planning and Organization	100 youths with Sickle Cell Disease (8–18 years old). Outpatient clinic.	Parent-proxy report (BRIEF-P)	EF significantly mediated the relationship between distraction and emotion-focused coping techniques on HRQL.
Mifflin, Chorney, and Dick (2016) ^1^	Attention, WM	13 females with chronic pain and 12 age- and gender-matched healthy youths. Heterogenous outpatient pain clinic sample.	Neuropsychological tests (unspecified)	Individuals with chronic pain had significantly lower WM scores than controls. Differences were found between groups on the most difficult selective attention task.
Turner, Wilcox, Nordstokke, Dick, Schroeder, and Noel (2021)	WM, Divided Attention, Inhibition, Planning, Shifting, Emotional Control	26 youths with chronic pain and their parents, 30 youths without chronic pain (13–17 yo). Heterogenous outpatient pain clinic sample.	Experimental performance-based tasks; IQ (WASI); Neuropsychological tests (e.g., Children’s Paced Auditory Serial Addition Test, Rey Complex Figure Test, Delis-Kaplan Executive Function System); BRIEF-2	Youth with chronic pain had significantly lower scores on several performance-based tests of WM, divided attention, inhibition, and flexibility. Statistically significant group differences on BRIEF-2 emotion control, shifting, task initiation/completion, WM, planning and organization.
Verhoeven, Dick, Eccleston, Goubert, and Crombez (2014)	Inhibitory Control, WM	164 school children (9–19 yo) without chronic pain. Experimental pain.	Performance-based tasks (Anti-saccade; Stroop; task switching); IQ (WISC)	Participants with better inhibition and WM performed a distraction task better; those with better switching abilities reported having paid more attention to the distraction task.
Weiss, Harbeck-Weber, Zaccariello, Kimondo, Harrison, and Bruce (2018)	Inhibition, Self-Monitoring, Shifting, Emotional Control, Task Completion, WM, Planning and Organization	41 youths with chronic pain (11–17 yo). Outpatient pain clinic and pain rehabilitation program.	Youth self-report (BRIEF-2); Neuropsychological tests (WRAML, TOMM)	Chronic pain sample demonstrated significant difficulties on at least one measure, with nine participants indicating difficulties on multiple measures.

^1^ The term “executive functioning” was not used, but domains of executive functioning were measured. WM = Working Memory. BRIEF-2 = Behavior Rating Inventory of Executive Function, 2nd Edition (Youth Self-Report). BRIEF-P = Behavior Rating Inventory of Executive Function–Parent. WISC = Wechsler Intelligence Scale for Children. K-BIT = Kaufman Brief Intelligence Test. WIAT = Wechsler Individual Achievement Test. WRAT = Wide Range Achievement Test. GORT = Grey Oral Reading Test. TOWL = Test of Written Language. TEA-Ch = Test of Everyday Attention for Children. WASI = Wechsler Abbreviated Scale of Intelligence. WRAML = Wide Range Assessment of Memory and Learning. TOMM = Test of Memory Malingering.

## Data Availability

Not applicable.
